# Effects of behavioural risk factors on high-cost users of healthcare: a population-based study

**DOI:** 10.17269/s41997-018-0127-5

**Published:** 2018-09-19

**Authors:** Amanda Alberga, Laura Holder, Kathy Kornas, Catherine Bornbaum, Laura Rosella

**Affiliations:** 1 Clinical Evaluative Sciences, Toronto, Canada; 2 Dalla Lana School of Public Health, The University of Toronto, Toronto, Canada; 3 Public Health Ontario, Toronto, ON Canada; 4 Dalla Lana School of Public Health, Health Sciences Bldg, 6th floor, 155 College St, Toronto, ON M5T 3M7 Canada

**Keywords:** Health promotion, Epidemiologic factors, Epidemiology, Administrative claims, healthcare, Health care costs, Promotion de la santé, Facteurs épidémiologiques, Épidémiologie, Données administratives des demandes de remboursement des soins de santé, Coûts des soins de santé

## Abstract

**Objectives:**

High-cost users (HCUs) are known to disproportionally incur the majority of healthcare utilization costs relative to their counterparts. A number of studies have highlighted the detrimental effects of risky health behaviours; however, only a few have demonstrated the link to HCUs, a meaningful endpoint for program and policy decision-makers. We investigated the association between health behaviour risks and downstream high-cost healthcare utilization.

**Methods:**

A combined cohort of participants from the Canadian Community Health Survey (CCHS) cycles 2005–2009 was linked to future population-based health administrative data in Ontario. Using person-centered costing methodology, CCHS respondents were ranked according to healthcare utilization costs and categorized as ever having HCU status in the 4 years following interview. Logistic regression models were used to estimate the association between various health behaviours on future HCU status.

**Results:**

Models estimated that smoking and physical inactivity were associated with a significant increase in the odds of becoming an HCU. Compared to individual behaviours, increasing the number of health behaviour risks significantly strengthened the odds of becoming an HCU in subsequent years.

**Conclusion:**

The analyses provide evidence that upstream health behaviours affect high-cost healthcare utilization. Health behaviours are a meaningful target for health promotion programs and policies. These findings can inform decision-makers on appropriate behavioural targets for those on an HCU trajectory and promote public health efforts to support healthcare system sustainability.

## Résumé

**Objectifs:**

Les utilisateurs à coût élevé (UCE) sont connus pour engendrer de manière disproportionnée la majorité des coûts d’utilisation des soins de santé par rapport à leurs congénères. Un certain nombre d’études ont souligné les effets nuisibles des comportements présentant des risques pour la santé; seulement quelques-unes ont cependant fait état d’un lien avec les UCE, ce qui est une importante considération finale pour les personnes qui décident des programmes et des politiques. Nous avons étudié l’association entre les risques dus aux comportements de santé et l’utilisation à coût élevé des soins de santé en aval.

**Méthode:**

Une cohorte combinant les participants des cycles 2005 à 2009 de l’Enquête sur la santé dans les collectivités canadiennes (ESCC) a été liée aux données administratives ultérieures sur la santé de la population en Ontario. À l’aide d’une méthode de calcul des coûts centrée sur la personne, nous avons classé les répondants de l’ESCC selon les coûts d’utilisation des soins de santé et mis à part ceux ayant eu un statut d’UCE au cours des 4 années suivant l’entretien pour l’ESCC. Des modèles de régression logistique ont servi à estimer l’association entre divers comportements de santé et le statut d’UCE ultérieur.

**Résultats:**

Selon les estimations des modèles, le tabagisme et la sédentarité sont associés à une hausse significative de la probabilité de devenir un UCE. Une comparaison des comportements individuels montre que l’accroissement du nombre de comportements présentant des risques pour la santé a renforcé de façon significative la probabilité de devenir un UCE au cours des années ultérieures.

**Conclusion:**

Les analyses démontrent que les comportements de santé en amont ont un effet sur l’utilisation à coût élevé des soins de santé. Les comportements de santé sont donc une cible valable pour les programmes et les politiques de promotion de la santé. Ces constatations peuvent éclairer les décideurs quant aux objectifs comportementaux pertinents pour les personnes qui sont sur une trajectoire d’UCE, et elles peuvent favoriser les démarches de santé publique à l’appui de la durabilité du système de soins de santé.

## Introduction

The high cost of healthcare utilization impacts health systems worldwide. In the context of improving healthcare system sustainability, there is a growing focus around a subset of individuals referred to as high-cost users (HCUs) who disproportionately incur a higher morbidity and thus a greater proportion of healthcare utilization costs when compared to their counterparts (Fitzpatrick et al. 2015; Rosella et al. 2014; Wodchis et al. 2016; Canadian Institute for Health Information 2015; Berk and Monheit 1992; Berk and Monheit 2001; Lemstra et al. 2009; Calver et al. 2006; Heslop et al. 2005; Radcliff et al. 2005; Reid et al. 2003; Rais et al. 2013; Roos et al. 2003; Roos et al. 1989; Kephart et al. 1998). Specifically, the top 5% of HCUs have been shown to account for virtually 50% of healthcare expenditures in both Canada and the US (Wodchis et al. 2016; Reid et al. 2003; Roos et al. 1989; Agency for Healthcare Research and Quality 2012; Ontario Association of Community Care Access Centres, Ontario Federation of Community Mental Health and Addiction Programs, & Ontario Hospital Association 2010).

To date, no study has explicitly focused on health behaviours and high-cost utilization, although several studies aimed at describing the characteristics of current HCUs have found that this subset of the population is typically older, often suffers from multiple chronic conditions, has lower socio-economic status, as well as engages in a number of risky health behaviours, such as smoking and physical inactivity (Fitzpatrick et al. 2015; Rosella et al. 2014; Wodchis et al. 2016). Among HCUs, chronic disease, infections and acute health events have been found to be a precursor to hospital admission, and about one third of HCUs remain an HCU in subsequent years (Wodchis et al. 2016). Recent studies have identified behavioural risk factors associated with hospitalization, prolonged hospital use and high-cost utilization (Rosella et al. 2014; Manuel et al. 2014; Manuel et al. 2016). Previous work has shown that 22% of health-related expenditures in Ontario are attributable to health behaviour risk factors such as physical inactivity and smoking, amounting to $4.9 billion in healthcare spending that could be prevented through the adoption of healthy behaviours (Manuel et al. 2016). Health behaviours seldom occur in isolation; however, research is limited on the impact that cumulative risky health behaviours have on perpetuating downstream high-cost utilization.

To inform public health policy and practice, an understanding of the upstream determinants that drive high healthcare utilization will help to inform appropriate preventive strategies for those who are on an HCU trajectory. Thus, the aim of this study was to utilize linked population and health administrative data from Ontario, Canada, to examine the effects of health behavioural risk factors on future HCU status.

## Methods

### Data sources

We conducted a retrospective cohort study of participants from three cycles of the Canadian Community Health Survey (CCHS) linked with population-based health administrative data from Ontario, Canada. Specifically, the cohort was composed of participants of CCHS cycle 3.1 (2005), cycle 4.1 (2007–2008) and cycle 5.1 (2009–2010). The CCHS is a cross-sectional survey administered by Statistics Canada that uses multi-stage sampling to collect demographic, socio-economic, health status and behavioural information from Canadians aged 12 years and older living in private dwellings. Comprehensive survey methodology for the CCHS is described elsewhere (Statistics Canada n.d.).

The Ontario Health Insurance Plan (OHIP) represents Ontario’s universal single-payer healthcare system, from which all healthcare-related encounters are recorded in health administrative databases. Annual healthcare spending was calculated using administrative data from all major sources of healthcare expenditures, including: physician services, inpatient hospitalizations, complex continuing care, emergency department visits, long-term care, prescriptions filled for those eligible for the Ontario Drug Benefit (ODB) program, home care, same-day surgery, inpatient rehabilitation, inpatient mental health, dialysis, oncology, outpatient services and non-physician services. To calculate valid healthcare spending costs, a person-centered costing methodology designed specifically for the Ontario population was utilized (Wodchis et al. 2012). Annual per-person costs for each of the 4 years following CCHS interview were calculated and ranked according to cost percentile. Costs were stratified according to HCU status and health behaviour risks (i.e., current smoker, physically inactive, very poor diet and binge drinking).

Participants were excluded if they could not be linked to administrative data (e.g., were not found in the Registered Persons Database), did not have a valid Ontario health card number during the observation window, were determined to be an HCU within 1 year of the CCHS interview date (i.e., the baseline year) or were less than 18 years of age. Only the first cycle of CCHS data was used for participants that appeared in multiple cycles.

### Outcome variable

Our outcome of interest was high-cost user (HCU) status 4 years following CCHS interview date. HCUs were defined as the persons in the top 5% of total annual healthcare utilization expenditures.

### Independent variables

Variable selection was guided by the Anderson-Newman Framework, a theoretical framework that articulates utilization based on predisposing factors, enabling factors and illness level (Andersen and Newman 2005).

Using a 2-year look back from CCHS interview date, prior utilization and comorbidity were captured according to Johns Hopkins Aggregated Diagnosis Groups (ADGs) version 10.0.1, a measure previously validated in the Ontario population (Austin et al. 2011).

Individual-level demographic, socio-economic and behavioural characteristics were attained from the CCHS. The cumulative effect of health behaviour risks was estimated by calculating the sum of the following health behavioural risk factors: current smoking (daily or occasional < 100 cigarettes in lifetime), binge drinking (≥ 5 drinks/occasion) in the last year, physical inactivity (average daily energy expenditure < 1.5 kcal/kg/day) and very poor diet (index score 0 to < 2). Diet is a point-based index (range: 0–10) derived from self-reported consumption of fruit and vegetables that has been previously validated for use (Manuel et al. 2014; Manuel 2012). Starting with 2 points per person, individuals receive points for each average daily serving of fruits and vegetables consumed. Two points were deducted for any of the following: high potato consumption, no carrot consumption, or excessive juice consumption, with negative final scores recorded as zero (Manuel et al. 2014).

### Statistical analysis

To account for the complexity of the CCHS survey design, ensure representative population estimates and account for survey non-response bias, Statistics Canada bootstrap survey weights were incorporated into the analysis using a pooled approach (Thomas and Wannell 2009). Approximately 83.6% of CCHS respondents were successfully linked to administrative data. Baseline descriptive statistics were calculated for HCUs (top 5%), non-HCUs (bottom 95%) and for the cohort overall. Logistic models were developed for 4-year trajectories among baseline non-HCUs to investigate associations between health behaviours according to unadjusted, age-sex-adjusted, age-sex-income adjusted and models adjusted for collapsed aggregated diagnostic groups (ADG)-age-sex and income. All analyses were conducted using SAS Enterprise version 9.4.

### Ethics approval

The study received ethics approval from the Research Ethics Board at the University of Toronto.

## Results

### Sample characteristics

Table 1 shows the baseline weighted distribution of socio-demographic characteristics for the sample. The sample included 87,338 adults aged 18 years or older, of whom 8.56% were classified as being HCUs at any point during the 4 years following CCHS interview. Compared to non-HCUs, HCUs were significantly more likely to be older, have lower household income, be overweight or obese and self-report low life stress. Relative to non-HCUs, a significantly greater proportion of HCUs reported being physically inactive and were less likely to report engaging in current smoking, binge drinking, poor diet and multiple risky health behaviours in the past year.

**Table 1:** Weighted distribution of demographic, socio-economic, health status and health behavioural characteristics among Ontario adults*

Characteristic	Overall % (95% CI)	HCU (top 5%) (95% CI)	Non-HCU (95% CI)	*p* value
Total population	*N* = 87,338	*N* = 7476	*N* = 79,862	
Socio-economics				
Sex				0.1555
Female	51.1% (51.0–51.3)	49.7% (47.6–51.7)	51.2% (51.1–51.4)	
Age (years)				< 0.0001
≤ 30	21.6% (21.4–21.7)	3.8% (3.0–4.6)	22.7% (22.5–22.8)	
30–39	18.0% (17.6–18.3)	3.7% (3.0–4.4)	18.9% (18.5–19.3)	
40–49	21.7% (21.1–22.3)	10.2% (8.5–11.8)	22.4% (21.8–23.0)	
50–59	17.4% (17.0–17.8)	17.4% (15.6–19.2)	17.4% (17.0–17.8)	
60–69	11.6% (11.3–11.9)	24.2% (22.5–25.8)	10.8% (10.5–11.2)	
70–79	6.7% (6.6–6.9)	22.8% (21.3–24.2)	5.8% (5.6–5.9)	
≥ 80	3.0% (2.9–3.1)	18% (16.7–19.4)	2.1% (1.9–2.2)	
Household income quintile (Equivalized)				< 0.0001
Quintile 1 (lowest 20%)	19.2% (18.7–19.8)	32.6% (30.5–34.6)	19.2% (18.7–19.8)	
Quintile 2	19.3% (18.8–19.8)	24.2% (22.2–26.2)	19.3% (18.8–19.8)	
Quintile 3	20.1% (19.7–20.6)	16.6% (15.1–18.1)	20.1% (19.7–20.6)	
Quintile 4	20.8% (20.3–21.3)	13.8% (12.3–15.2)	20.8% (20.3–21.3)	
Quintile 5 (highest 20%)	20.5% (20.0–21.1)	12.9% (11.4–14.4)	20.5% (20.0–21.1)	
Health status				
Body mass index (kg/m^2^)				< 0.0001
Underweight, BMI < 18.5	2.6% (2.4–2.8)	2.3% (1.7–2.9)	2.6% (2.4–2.8)	
Normal weight, BMI 18.5–24.9	45.5% (44.9–46.1)	37.1% (35.1–39.1)	46.0% (45.4–46.6)	
Overweight, BMI 25–29.9	34.1% (33.6–34.7)	37.6% (35.4–39.8)	33.9% (33.3–34.5)	
Moderately obese, BMI 30–34.9	12.0% (11.6–12.4)	15.5 (13.9–17.0)	11.8% (11.4–12.2)	
Very obese, BMI 35–39.9	3.2% (3.0–3.4)	4.8% (3.9–5.7)	3.1% (3.0–3.3)	
Severely obese, BMI > 40	2.5% (2.3–2.7)	2.8% (2.2–3.4)	2.5% (2.3–2.7)	
Life stress				
Low (A bit, not very, none)	76.7% (76.2–77.2)	79.1% (77.3–80.9)	76.5% (76.0–77.0)	
Health behaviours				
Smoking status				
Never (never smoked a whole cigarette)	40.3% (39.7–40.9)	33.0% (30.7–35.2)	40.8% (40.2–41.4)	< 0.0001
Former (former daily or former occasional)	38.2% (37.6–38.8)	47.2% (45.2–49.3)	37.7% (37.1–38.3)	
Current smoker (daily or occasional)	21.4% (21.0–21.9)	19.8% (18.2–21.4)	21.5% (21.1–22.0)	
Alcohol consumption (past year)				
Regular drinker (1 or more drinks per week)	20.1% (19.6–20.6)	27.6% (25.6–29.7)	19.6% (19.2–20.1)	< 0.0001
Occasional drinker (< 1 drink/month)	15.3% (14.9–15.7)	19.1% (17.3–20.9)	15.1% (14.7–15.5)	
Binge drinker (≥ 5 drinks/occasion)	41.6% (40.9–42.2)	20.0% (18.3–21.6)	42.9% (42.3–43.6)	
Current non-drinker (no consumption in last 12 months)	23.0% (22.4–23.6)	33.3% (31.1–35.5)	22.4% (21.7–23.0)	
Daily physical activity				
Inactive (< 1.5 kcal/kg/day)	49.8% (49.2–50.4)	59.6% (57.4–61.8)	49.2% (48.6–49.9)	< 0.0001
Moderately active (1.5 to 2.9 kcal/kg/day)	24.6% (24.1–25.1)	22.5% (20.8–24.3)	24.8% (24.2–25.3)	
Active (3.0 kcal/kg/day)	25.6% (25.0–26.1)	17.9% (16.3–19.5)	26.0% (25.5–26.6)	
Diet				
Very poor (index score 0 to < 2)	11.9% (11.5–12.3)	10.5% (9.4–11.7)	12.0% (11.6–12.4)	0.0500
Fair (index score 2 to < 4)	36.4% (35.8–37.0)	37.8% (35.9–39.8)	36.3% (35.7–36.9)	
Adequate (index score 4 to 10)	51.7% (51.1–52.2)	51.6% (49.5–53.7)	51.7% (51.1–52.3)	
Health behaviour risks				
No behavioural risks	24.1% (23.6–24.6)	29.1% (27.2–30.9)	23.8% (23.3–24.3)	< 0.0001
1 behavioural risk	44.2% (43.7–44.8)	46.5% (44.3–48.7)	44.1% (43.5–44.7)	
2 behavioural risks	22.1% (21.7–22.6)	17.7% (16.2–19.2)	22.4% (21.9–22.9)	
3 behavioural risks	8.0% (7.7–8.3)	5.7% (4.8–6.5)	8.2% (7.8–8.5)	
4 behavioural risks	1.5% (1.4–1.6)	1.1% (0.7–1.5)	1.6% (1.4–1.7)	

*Weighted using CCHS bootstrap weights to provide population estimates

### Distribution of healthcare spending

Table 2 presents weighted average per-person costs stratified by risky health behaviours. On average, individuals who reported physical inactivity incurred the highest healthcare costs in both HCUs and non-HCUs, followed by those who reported poor diet, smoking and binge drinking behaviours (Table 2). Comparatively, across all risky behaviours, healthcare costs were about 12 to 16 times higher for HCUs than for non-HCUs. Among those who engaged in risky behaviours, the highest costs incurred by HCUs were linked to inpatient hospitalizations, whereas the highest costs incurred by non-HCUs corresponded to prescription drugs through the Ontario Drug Benefit (ODB) program.

**Table 2:** Weighted average per-person expenditure* stratified by health behavioural risks

Health behaviours	Current smoker (95% CI)	Physically inactive (95% CI)	Very poor diet (95% CI)	Binge drinker (95% CI)
Overall				
Service type				
Physician services	$262 (255–269)	$319 (313–324)	$246 (237–255)	$1831 (1794–1868)
Hospitalizations	$1609 (1441–1776)	$2113 (1972–2254)	$1588 (1293–1882)	$1037 (960–1114)
Complex continuing care	$105 (29–181)	$127 (90–165)	$102 (41–164)	$45 (6–83)
Emergency department	$431 (413–449)	$384 (373–392)	$401 (381–422)	$314 (304–323)
Long-term care	$66 (34–98)	$162 (135–190)	$109 (45–173)	$15 (8–22)
Ontario Drug Benefit (ODB)	$936 (847–1026)	$1317 (1253–1380)	$1039 (908–1171)	$382 (342–421)
Home care	$236 (194–277)	$376 (345–406)	$230 (190–271)	$100 (88–113)
Same-day surgery	$353 (334–372)	$423 (407–439)	$354 (324–385)	$306 (293–320)
Rehabilitation	$63 (41–84)	$119 (100–139)	$83 (54–113)	$39 (22–57)
Non-physician services	$25 (23–28)	$58 (55–62)	$35 (30–39)	$19 (18–21)
All services	$6859 (6515–7202)	$8932 (8537–9328)	$6835 (6346–7324)	$4653 (4482–4824)
Top 5% high-cost users				
Service type				
Physician services	$362 (331–393)	$510 (484–536)	$405 (346–464)	$10,041 (9439–10,643)
Hospitalizations	$20,507 (17,888–23,125)	$22,591 (20,883–24,299)	$22,300 (17,126–27,473)	$21,000 (19,015–22,985)
Complex continuing care	$1945 (540–3351)	$1896 (1340-2542)	$2089 (836–3341)	$1580 (231–2929)
Emergency department	$1762 (1617–1907)	$1664 (1582–1746)	$1609 (1468–1749)	$1449 (1342–1558)
Long-term care	$1222 (631–1814)	$2401 (1986–2816)	$2189 (889–3489)	$530 (276–785)
Ontario Drug Benefit (ODB)	$6536 (5472–7601)	$7151 (6518–7783)	$7247 (5767–8726)	$2266 (2156–6689)
Home care	$2787 (2155–3420)	$8190 (6042–10,338)	$2827 (2208–3446)	$2002 (1656–2348)
Same-day surgery	$1338 (1162–1514)	$1369 (1253–1485)	$1530 (1252–1809)	$1639 (1445–1833)
Rehabilitation	$1150 (757–1543)	$1775 (1483–2066)	$1696 (1112–2279)	$1370 (769–1972)
Non-physician services	$105 (73–136)	$260 (233–287)	$203 (128–279)	$99 (79–118)
All services	$54,927 (51,208–58,646)	$61,753 (57,053–66,454)	$57,050 (50,388–63,712)	$52,039 (48,572–55,507)
Non high-cost users				
Service type				
Physician services	$256 (249–263)	$305 (299–311)	$238 (229–247)	$1591 (1555–1627)
Hospitalizations	$533 (497–568)	$642 (611–673)	$522 (471–574)	$454 (428–479)
Complex continuing care	$0–	$0–	$0–	$0–
Emergency department	$355 (340–370)	$292 (284–301)	$339 (320–358)	$208 (272–289)
Long-term care	$0–	$1 (0–2)	$2 (0–5)	$0–
Ontario Drug Benefit (ODB)	$618 (553–682)	$897 (853–942)	$720 (610–830)	$262 (232–293)
Home care	$90 (75–106)	$125 (111–139)	$97 (72–121)	$45 (38–51)
Same-day surgery	$297 (280–313)	$355 (340–370)	$294 (267–321)	$268 (256–280)
Rehabilitation	$0–	$0–	$0–	$0–
Non-physician services	$21 (19–23)	$44 (41–47)	$26 (23–29)	$17 (16–19)
All services	$4122 (3985–4258)	$5137 (5035–5240)	$4252 (4048–4456)	$3268 (3190–3347)

*Expenditure calculated in Canadian dollars ($CAD) for each of the 4 years following CCHS interview

### Odds of high-cost user status

Table 3 presents unadjusted and adjusted logistic models examining the odds of becoming an HCU in the 4 years following a CCHS interview based on the selected health behaviour indicators.

**Table 3:** Weighted unadjusted and adjusted odds of being a high-cost user (top 5%) in 4 years post CCHS interview

Characteristic	Odds of being an HCU (top 5%)				
	Unadjusted (95% CI)	Age-sex-adjusted (95% CI)	Age-sex-income-adjusted (95% CI)	Fully-adjusted* (95% CI)	*p* value
Smoking status					< 0.0001
Never (never smoked a whole cigarette)	ref ((Fitzpatrick et al. 2015).00)	ref ((Fitzpatrick et al. 2015).00)	ref ((Fitzpatrick et al. 2015).00)	ref ((Fitzpatrick et al. 2015).00)	
Former (former daily or former occasional)	1.55 (1.39–1.72)	1.15 (1.02–1.30)	1.19 (1.04–1.36)	1.12 (0.98–1.28)	
Current smoker (daily or occasional)	1.14 (0.99–1.29)	1.67 (1.43–1.95)	1.63 (1.37–1.95)	1.61 (1.35–1.92)	
Alcohol consumption (past year)					0.0741
Regular drinker (1 or more drinks per week)	ref ((Fitzpatrick et al. 2015).00)	ref ((Fitzpatrick et al. 2015).00)	ref ((Fitzpatrick et al. 2015).00)	ref ((Fitzpatrick et al. 2015).00)	
Occasional drinker (< 1 drink/month)	0.89 (0.78–1.04)	1.29 (1.19–1.53)	1.13 (0.96–1.35)	1.12 (0.93–1.33)	
Binge drinker (≥ 5 drinks/occasion)	0.33 (0.29–0.36)	0.91 (0.78–1.08)	0.86 (0.74–1.07)	0.89 (0.76–1.04)	
Current non-drinker (no consumption in last 12 months)	1.06 (0.93–1.20)	1.36 (1.19–1.57)	1.13 (0.98–1.32)	1.09 (0.94–1.25)	
Physical activity					0.0003
Inactive (< 1.5 kcal/kg/day)	1.76 (1.56–1.99)	1.42 (1.25–1.61)	1.34 (1.17–1.52)	1.27 (1.12–1.45)	
Moderately active (1.5 to 2.9 kcal/kg/day)	1.32 (1.16–1.51)	1.09 (0.96–1.26)	1.08 (0.94–1.24)	1.06 (0.92–1.23)	
Active (3.0 kcal/kg/day)	ref ((Fitzpatrick et al. 2015).00)	ref ((Fitzpatrick et al. 2015).00)	ref ((Fitzpatrick et al. 2015).00)	ref ((Fitzpatrick et al. 2015).00)	
Diet					0.2645
Very poor (index score 0 to < 2)	0.87 (0.77–0.99)	1.31 (1.13–1.53)	1.16 (0.98–1.37)	1.13 (0.95–1.34)	
Fair (index score 2 to < 4)	1.04 (0.95–1.14)	1.14 (1.03–1.26)	1.09 (0.98–1.22)	1.08 (0.97–1.21)	
Adequate (index score 4 to 10)	ref ((Fitzpatrick et al. 2015).00)	ref ((Fitzpatrick et al. 2015).00)	ref ((Fitzpatrick et al. 2015).00)	ref ((Fitzpatrick et al. 2015).00)	
Health behaviour risks					0.0067
1 behavioural risk	0.86 (0.78–0.96)	1.14 (1.02–1.28)	1.10 (0.98–1.24)	1.08 (0.96–1.21)	
2 behavioural risks	0.65 (0.57–0.73)	1.33 (1.16–1.53)	1.29 (1.11–1.49)	1.26 (1.09–1.47)	
3 behavioural risks	0.57 (0.48–0.68)	1.48 (1.22–1.79)	1.38 (1.12–1.69)	1.37 (1.11–1.69)	
4 behavioural risks	0.58 (0.39–0.85)	1.76 (1.18–2.63)	1.29 (0.82–2.04)	1.28 (0.81–2.03)	
No behavioural risks	ref ((Fitzpatrick et al. 2015).00)	ref ((Fitzpatrick et al. 2015).00)	ref ((Fitzpatrick et al. 2015).00)	ref ((Fitzpatrick et al. 2015).00)	

*Model adjusted for age, sex, income and ADGs^a^

^a^Johns Hopkins ADGs are a clinical measure of morbidity

Of the health behaviours examined, unadjusted and adjusted logistic regression models revealed that smoking status and physical inactivity were most strongly associated with high-cost utilization. In the fully adjusted models, the association between being a former smoker and HCU was attenuated, while the strength of association between being a current smoker and HCU increased compared to the unadjusted model (AOR: 1.61; 95% CI: 1.35–1.92; *p* < 0.0001). The odds of being an HCU were not significantly different for binge drinkers relative to regular drinkers (AOR: 0.89; 95% CI: 0.76–1.04; *p* = 0.1278). Compared to participants who were physically active, respondents who reported being inactive had 27% greater odds of becoming an HCU (AOR: 1.27; 95% CI: 1.12–1.45; *p* = 0.0003). Relative to individuals who consumed an adequate diet, those who consumed a very poor diet had 31% higher odds (AOR: 1.31; 95% CI: 1.13–1.53; *p* = 0.001) of becoming an HCU in the age-sex-adjusted model, though this result was not significant in the fully adjusted model (AOR: 1.13; 95% CI: 0.95–1.34; *p* = 0.2645). Relative to engaging in no risky health behaviours, as the cumulative number of existing health behaviour risks increased, the odds of becoming an HCU also increased. The strongest association was found among individuals with three behavioural risk factors who had a 37% increase in the odds of becoming an HCU (AOR: 1.37; 95% CI: 1.11–1.69; *p* = 0.0034), whereas the association between having four behavioural risk factors and becoming an HCU was not significant in the fully adjusted model (AOR: 1.28; 95% CI: 0.81–2.03; *p* = 0.2869) in the 4 years following CCHS interview.

In the sensitivity analysis, adjusting for Johns Hopkins ADGs using a summarized ADG-score compared to a grouped ADG-adjusted model had little to no effect on the model estimates, suggesting the robustness in both measures when adjusting for aggregated diagnoses in regression modeling.

## Discussion

This longitudinal population-based study quantified the extent to which a range of health behaviours were associated with future HCU status in Ontario. Our findings demonstrate that smoking and physical inactivity are prominent upstream health behaviours linked to the odds of becoming an HCU within 4 years, in alignment with current evidence linking unhealthy behaviours to hospitalization and high-cost healthcare users (Rosella et al. 2014; Manuel et al. 2014; Manuel et al. 2016). Our findings also extend previous research suggesting a cumulative impact of socio-economic position and health behaviours on healthcare use (Manuel et al. 2016). To our knowledge, this study is the first to quantify the impact of cumulative risk behaviours in relation to healthcare utilization among the top 5% of HCUs. Our findings demonstrate a gradient towards an HCU trajectory when accounting for the real-world circumstances in which risk behaviours and social inequities co-exist. While some health behaviours such as smoking and heavy drinking foreshadow future population health and health system-related burden, others such as physical activity and fruit and vegetable consumption are health-promoting behaviours, thus highlighting the value of capturing the cumulative impact.

Our findings revealed that within the 4-year trajectory examined, smoking, physical inactivity and engaging in multiple risky health behaviours were significantly linked to HCU status. Conversely, in the short term, we did not find having an unhealthy diet (measured using fruit and vegetable consumption) or alcohol consumption to be associated with future HCU status. When stratifying per-person health sector expenditures according to health behavioural risks, we demonstrated the highest cost burden related to physical inactivity in both HCUs and non-HCUs, in which HCUs have the highest spending within hospitalization and home care sectors. Health promotion and prevention strategies aimed at reducing the burden of physical inactivity at the population level, which in turn mediate the HCU trajectory, would have a strong impact on the downstream health and health system consequences. Our results coincide with previous research which found that an estimated 53% of healthcare costs ($47.3 billion) attributable to physical inactivity could be avoided through improved policy and program interventions specifically targeted at physical inactivity within the population (Manuel et al. 2016).

Our findings confirmed that current smoking status is associated with becoming a HCU in subsequent years. Previous research from Ontario has also shown that, compared to non-smokers, heavy smokers have the highest hospital use, some of the highest attributable healthcare costs, a reduced life expectancy by almost 12 years and high risk of premature death (Manuel et al. 2014; Manuel et al. 2016; Manuel 2012). Our findings highlight the continued importance of appropriate tobacco control strategies to curb downstream high-cost healthcare utilization, and the need for public health measures concerning tobacco dependence and cessation for those on an HCU trajectory.

Physical inactivity was found to be associated with future HCU status. Within the literature, physical inactivity has been previously linked to reduced life expectancy and prolonged hospital stay (Manuel et al. 2014; Manuel 2012). Our findings suggest that those on an HCU trajectory may benefit from lifestyle interventions focused on promoting physical activity.

The relative impact of diet on HCU status was attenuated in models adjusted for income and CADGs, though prior research has found that food insecurity, a factor linked to diet, increased the odds of future HCU status (Fitzpatrick et al. 2015). It is plausible that the relationship between diet and HCU status is largely driven by factors related to socio-economic position. Socio-economic factors such as income may modify the relative effect of health behaviours, as demonstrated by a change in the magnitude of effect when income was added to health behaviour models. For example, individuals on the HCU trajectory may be less likely to engage in healthy behaviours primarily due to socio-economic constraints. It is important to recognize that the combination of behaviours that make up a healthy lifestyle are multidimensional and influenced by a number of physical, social and environmental stimuli (Peel Public Health 2012). We were unable to capture important components of poor diet such as low fibre, high sodium or trans fats intake in our diet index—it is possible that a more robust measure would yield a significant effect. Alcohol consumption is associated with a number of medical conditions such as cancer, high blood pressure, liver disease and depression (Corrao et al. 2004; U.S. Department of Health and Human Services 2000). It is plausible that individuals on the HCU trajectory previously engaged in alcohol consumption, but perhaps altered their drinking habits to prevent further medical complications (Rosella et al. 2014).

The current findings build on our understanding of how health behaviours can perpetuate future high resource utilization and emphasize that health behaviours are important determinants of future healthcare utilization. In all fully-adjusted models, the negative impact of engaging in multiple risky health behaviours on healthcare utilization was apparent by the clear incremental gradient in HCU status and cumulative number of health behavioural risk factors. Notably, individuals with multiple unhealthy behaviours were the least likely to consult with their general practitioner (Feng et al. 2014), which may exacerbate the HCU trajectory. Our findings underscore the need for an integrated approach to prevention that addresses both behavioural and population-level factors to reduce the likelihood of becoming a future HCU.

### Limitations

The results of this study highlight the negative implications of upstream risky health behaviours and their role in perpetuating high health resource utilization. Longitudinal analyses using data linkages at the individual level, a large sample size as well as representative population-based surveys are inherent strengths of the study. Nevertheless, there are important limitations that must be acknowledged.

Due to availability of health administrative data during the time period the analysis was being conducted, we accessed data for all health services needed for the costing methodology up to a maximum follow-up period of 4 years from CCHS interview date, and therefore did not capture risk associated with health behaviours over longer periods of time. Furthermore, we are only able to capture health behaviours at a single point in time and thus unable to account for behaviour changes before or after the survey date. Investigating high-cost user trajectories in relation to lifestyle patterns or changes over time requires longitudinal data with repeated measures over time, which is quite limited; however, would be interesting as a future area of study. In addition, we did not use a known gold standard for dietary behaviour (24-h diet recall), which may have caused a dilution of the effects in the event of recall error. Self-reported health behaviour measures may be more subject to social desirability bias, in which participants are less likely to report health behaviours perceived as undesirable. Our findings related to diet and alcohol are consistent with previous studies where self-reported diet and alcohol exposure have broadly resulted in an underestimation of risk (Manuel et al. 2016; Whitford et al. 2009; Shields et al. 2008; Wong et al. 2012; Muggah et al. 2013).

Informal (e.g., family/friend caregiving costs) and formal healthcare operating, capital, individual copayments (e.g., prescription drug), private health insurance arranged services and community-based healthcare expenditures such as ambulatory, ophthalmology and orthopedic care are not included in the costing methodology and thus were not accounted for. While the costing methodology is robust, hospital admission dates may predate CCHS interview dates, and discharge may occur after the costing period specified, thus resulting in underestimated individual costs (e.g., longer length of stay in hospital which began before CCHS interview and extended beyond the costing period).

The CCHS sampling frame includes individuals living in private dwellings only, thus homeless individuals, First Nations people living on reserve, as well as institutionalized individuals were excluded, which obscures our ability to generalize our results to important populations at risk, who may have greater health needs and a higher likelihood of health behaviour risk factors. As a result of the sampling frame, the magnitude of our estimated effects likely underestimate high-cost healthcare utilization associated with population health behaviours to the same extent that CCHS respondents are healthier (Keyes et al. 2018). Furthermore, despite its relevance as a health behaviour, we were unable to examine the impact of sleep, given that information on sleep is only captured in more recent versions of the CCHS. Insufficient sleep has been previously linked to chronic disease, obesity and mental illness, and therefore is an important area for future high health resource utilization research (Liu et al. 2013; Chapman et al. 2013). Last, when multiple comparisons are made by considering a set of statistical inferences simultaneously, there is an increased possibility of a false-positive finding (type 1 error); nevertheless, our findings align with the published literature, thus enhancing confidence in our results (Fitzpatrick et al. 2015; Rosella et al. 2014; Manuel et al. 2014; Manuel et al. 2016; Rothman 1990).

## Conclusion

Our study builds on previous work investigating the association between high healthcare utilization and behavioural factors. Our findings support that, in the short term, smoking status and physical activity are relevant behavioural targets for those on an HCU trajectory. Additionally, our findings suggest that strategies aimed at mitigating high healthcare utilization should consider the dynamic interrelationships between health behaviours and socio-economic position and may consequently require the integration of both individual- and population-level prevention approaches.

## References

[CR1] Agency for Healthcare Research and Quality. The concentration and persistence in the level of health expenditures over time: estimates for the US population, 2008–2009. Rockville: Agency for Healthcare Research and Quality, Survey MEP; 2012.

[CR2] ANDERSEN RONALD, NEWMAN JOHN F. (2005). Societal and Individual Determinants of Medical Care Utilization in the United States. Milbank Quarterly.

[CR3] Austin PC, van Walraven C, Wodchis WP, Newman A, Anderson GM (2011). Using the Johns Hopkins aggregated diagnosis groups (ADGs) to predict mortality in a general adult population cohort in Ontario, Canada. Medical Care.

[CR4] Berk ML, Monheit AC (1992). The concentration of health expenditures: An update. Health Affairs.

[CR5] Berk ML, Monheit AC (2001). The concentration of health care expenditures, revisited. Health Affairs.

[CR6] Calver J, Brameld KJ, Preen DB, Alexia SJ, Boldy DP, McCaul KA (2006). High-cost users of hospital beds in Western Australia: A population-based record linkage study. Medical Journal of Australia.

[CR7] Canadian Institute for Health Information. National Health Expenditure Trends, 1975 to 2015 report. Toronto: Canadian Institute for Health Information; 2015.

[CR8] Chapman, D. P., Presley-Cantrell, L. R., Liu, Y., Perry, G. S., Wheaton, A. G., & Croft, J. B. (2013). Frequent insufficient sleep and anxiety and depressive disorders among US community dwellers in 20 states, 2010. *Psychiatric Services*. 10.1176/appi.ps.201200226 23543168

[CR9] Corrao G, Bagnardi V, Zambon A, La Vecchia C (2004). A meta-analysis of alcohol consumption and the risk of 15 diseases. Preventive Medicine.

[CR10] Feng X, Girosi F, McRae IS (2014). People with multiple unhealthy lifestyles are less likely to consult primary healthcare. BMC Family Practice.

[CR11] Fitzpatrick T, Rosella LC, Calzavara A, Petch J, Pinto AD, Manson H (2015). Looking beyond income and education: Socioeconomic status gradients among future high-cost users of health care. American Journal of Preventive Medicine.

[CR12] Heslop L, Athan D, Gardner B, Diers D, Poh BC (2005). An analysis of high-cost users at an Australian public health service organization. Health Services Management Research.

[CR13] Kephart G, Thomas VS, MacLean DR (1998). Socioeconomic differences in the use of physician services in Nova Scotia. American Journal of Public Health.

[CR14] Keyes KM, Rutherford C, Popham F, Martins SS, Gray L (2018). How healthy are survey respondents compared with the general population?: Using survey-linked death records to compare mortality outcomes. Epidemiology.

[CR15] Lemstra, M., Mackenbach, J., Neudorf, C., & Nannapaneni, U. (2009). High health care utilization and costs associated with lower socio-economic status: Results from a linked dataset. *Canadian Journal of Public Health*, 180–183. 10.1007/BF03405536 PMC6973662 19507718

[CR16] Liu Y, Croft JB, Wheaton AG, Perry GS, Chapman DP, Strine TW (2013). Association between perceived insufficient sleep, frequent mental distress, obesity and chronic diseases among US adults, 2009 behavioral risk factor surveillance system. BMC Public Health.

[CR17] Manuel DG. Seven more years: The impact of smoking, alcohol, diet, physical activity and stress on health and life expectancy in Ontario: An ICES/PHO report: Institute for Clinical Evaluative Sciences; 2012.

[CR18] Manuel DGPR, Benne C, Laporte A, Wilton AS, Gandhi S, Yates EA, Henry DA (2016). A $4.9 billion decrease in health care expenditure: The ten-year impact of changing smoking, alcohol, diet and physical activity on health care use in Ontario.

[CR19] Manuel DG, Perez R, Bennett C, Rosella LCA, Choi BC. 900,000 days in hospital: the annual impact of smoking, alcohol, diet and physical activity on hospital use in Ontario 2014.

[CR20] Muggah E, Graves E, Bennett C, Manuel DG (2013). Ascertainment of chronic diseases using population health data: A comparison of health administrative data and patient self-report. BMC Public Health.

[CR21] Ontario Association of Community Care Access Centres, Ontario Federation of Community Mental Health and Addiction Programs, & Ontario Hospital Association. Ideas and opportunities for bending the health care cost curve. Scarborough: Ontario Association of Community Care Access Centres; Ottawa: Ontario Federation of Community Mental Health and Addiction Programs; Toronto: Ontario Hospital Association; 2010. p. 2.

[CR22] Peel Public Health. Changing Course: Creating Supportive Environments for Healthy Living in Peel. Toronto, Ontario: Peel Public Health, 2012.

[CR23] Radcliff TA, Côté MJ, Duncan RP (2005). The identification of high-cost patients. Hospital Topics.

[CR24] Rais S, Nazerian A, Ardal S, Chechulin Y, Bains N, Malikov K (2013). High-cost users of Ontario's healthcare services. Healthcare Policy.

[CR25] Reid R, Evans R, Barer M, Sheps S, Kerluke K, McGrail K (2003). Conspicuous consumption: Characterizing high users of physician services in one Canadian province. Journal of Health Services Research & Policy.

[CR26] Roos Noralou P., Shapiro Evelyn, Tate Robert (1989). Does a Small Minority of Elderly Account for a Majority of Health Care Expenditures?: A Sixteen-Year Perspective. The Milbank Quarterly.

[CR27] Roos Noralou, Burchill Charles, Carriere Keumhee (2003). Who are the High Hospital Users? A Canadian Case Study. Journal of Health Services Research & Policy.

[CR28] Rosella LC, Fitzpatrick T, Wodchis WP, Calzavara A, Manson H, Goel V (2014). High-cost health care users in Ontario, Canada: Demographic, socio-economic, and health status characteristics. BMC Health Serv Res.

[CR29] Rothman Kenneth J. (1990). No Adjustments Are Needed for Multiple Comparisons. Epidemiology.

[CR30] Shields M, Gorber SC, Tremblay MS (2008). Estimates of obesity based on self-report versus direct measures. Health Reports.

[CR31] Statistics Canada. Canadian Community Health Survey Annual Component [Available from: http://www23.statcan.gc.ca/imdb/p2SV.pl?Function=getSurvey&SDDS=3226.

[CR32] Thomas S, Wannell B (2009). Combining cycles of the Canadian community health survey. Health Reports.

[CR33] U.S. Department of Health and Human Services (2000). Tenth special report to the U.S. congress on alcohol and health.

[CR34] Whitford JL, Widner SC, Mellick D, Elkins RL (2009). Self-report of drinking compared to objective markers of alcohol consumption. The American Journal of Drug and Alcohol Abuse.

[CR35] Wodchis W, Bushmeneva K, Nikitovic M, McKillop I (2012). Guidelines on person-level costing using administrative databases in Ontario.

[CR36] Wodchis Walter P., Austin Peter C., Henry David A. (2016). A 3-year study of high-cost users of health care. Canadian Medical Association Journal.

[CR37] Wong SL, Shields M, Leatherdale S, Malaison E, Hammond D (2012). Assessment of validity of self-reported smoking status. Health Reports.

